# Dim artificial light at night reduces the cellular immune response of the black field cricket, *Teleogryllus commodus*

**DOI:** 10.1111/1744-7917.12665

**Published:** 2019-03-07

**Authors:** Joanna Durrant, Mark P. Green, Therésa M. Jones

**Affiliations:** ^1^ The School of BioSciences, Faculty of Science University of Melbourne Victoria 3010 Australia

**Keywords:** circadian rhythm, ecophysiology, immune function, immunomodulation, invertebrate, light pollution, melatonin, urbanization

## Abstract

A functioning immune system is crucial for protection against disease and illness, yet increasing evidence suggests that species living in urban areas could be suffering from immune suppression, due to the presence of artificial light at night (ALAN). This study examined the effects of ecologically relevant levels of ALAN on three key measures of immune function (haemocyte concentration, lytic activity, and phenoloxidase activity) using a model invertebrate species, the Australian black field cricket, *Teleogryllus commodus*. We reared crickets under an ecologically relevant daily light‐cycle consisting of 12 hr bright daylight (2600 lx) followed by either 12 h darkness (0 lx) or dim environmentally relevant ALAN (1, 10, 100 lx), and then assessed immune function at multiple time points throughout adult life using haemolymph samples. We found that the presence of ALAN had a clear negative effect on haemocytes, while the effects on lytic activity and phenoloxidase activity were more complex or largely unaffected by ALAN. Furthermore, the effects of lifelong exposure to ALAN of 1 lx were comparable to those of 10 and 100 lx. Our data suggest that the effects of ALAN could be large and widespread, and such reductions in the core immune response of individuals will likely have greater consequences for fitness and survival under more malign conditions, such as those of the natural environment.

## Introduction

The global presence of artificial light at night (ALAN) is increasing rapidly in terms of its intensity and distribution making it one of the most pervasive recent forms of anthropogenic pollution (Bennie *et al*., 2015; Gaston *et al*., 2015; Falchi *et al*., 2016). The ecological consequences of ALAN are demonstrated across a range of taxa and ecological levels (Rich & Longcore, 2006; Gaston *et al*., 2014; Henneken & Jones, 2017; Tierney *et al*., 2017; Hopkins *et al*., 2018). A growing body of evidence links the presence of ALAN with physiological effects including altered development (Bruning *et al*., 2011; van Geffen *et al*., 2014; Raap *et al*., 2016a; Durrant *et al*., 2018), survival (McLay *et al*., 2017), aging (Vinogradova *et al*., 2009), shifts to the timing of reproductive events (Dominoni *et al*., 2013a,c; Robert *et al*., 2015; Brüning *et al*., 2016; Botha *et al*., 2017), and increased risk of disease and illness, including cancer (Hastings *et al*., 2003; Stevens, 2009; Bedrosian *et al*., 2011a; Fonken *et al*., 2013; Borniger *et al*., 2014; Smolensky *et al*., 2015; Dominoni *et al*., 2016). Correlated with (and potentially underpinning) these physiological impacts are the largely negative effects of ALAN on immune function demonstrated in birds and mammals, including Japanese quail (Moore & Siopes, 2000), rats (Oishi *et al*., 2006; Cisse *et al*., 2017), Siberian hamsters (Aubrecht *et al*., 2014), blackbirds (Russ *et al*., 2015a), and great tits (Raap *et al*., 2016b; Ouyang *et al*., 2017).

The immune system is critical for defending an organism against insult from bacterial and viral infections, as well as other diseases or damage, and as such is a crucial component of individual fitness (Schmid‐Hempel, 2005). Most studies to date, including those highlighted above, are vertebrate focused and typically immune function is assayed at a single time point; longitudinal studies exploring the effect of ecological relevant levels of ALAN at the level of the individual are rare. Invertebrates, and particularly insects, offer an attractive opportunity to address some of these gaps in our current understanding of the effects of ALAN on invertebrates immune function, and, in particular, the long‐term effects throughout an entire lifetime. Invertebrates lack an adaptive immune system but their innate immune system is highly effective and has cellular recognition and defensive pathways that are analogous with vertebrates (for a comprehensive coverage of the discipline see Rosales, 2017). The immune system of invertebrates includes immune cells, as well as chemicals, enzymes and antimicrobial peptides in body fluids (Cooper & Lemmi, 1981; Beck & Habicht, 1996; Murphy & Weaver, 2016). These systems interact to mount an effective immune response (Siva‐Jothy *et al*., 2005; Haine *et al*., 2008).

Three common measures are used to explore variation in insect immunocompetence: (i) the concentration of circulating haemocytes provides a measure of the capacity for an individual to mediate the core cellular defense pathway, including phagocytosis and encapsulation (Ribeiro & Brehélin, 2006); (ii) lysozyme‐like (herein referred to as lytic) activity provides an indication of the capacity of antibacterial enzymes principally involved in degrading bacterial cell walls to resist a bacterial challenge (Beckage, 2008); and (iii) phenoloxidase (PO), which becomes activated upon cuticular wounding or infection and is an important precursor involved in repair and encapsulation following infection or parasitism of larger foreign bodies (Kanost & Gorman, 2008).

In this study, we explored patterns of immune activity following lifelong exposure to varying levels of ALAN in the nocturnal Australian black field cricket, *Teleogryllus commodus*. The black field cricket is an ideal species to explore ALAN related questions. It is a native inhabitant of grassland areas in Australia, but it is also present in urban environments and thus it is likely directly exposed to the presence of ALAN (Otte & Alexander, 1983). Previous research demonstrates that exposure to ecologically relevant levels of ALAN (1, 10, and 100 lx) consistently prolongs the juvenile developmental period, resulting in increased adult size (Durrant *et al*., 2018) and affects some aspects of reproduction (Botha *et al*., 2017). *Teleogryllus commodus* is a model species for invertebrate immune studies (Simmons *et al*., 2005; Simmons *et al*., 2010; Bailey *et al*., 2011; Drayton & Jennions, 2011; Dowling & Simmons, 2012; Drayton *et al*., 2012; McNamara *et al*., 2014) and exposure during development to constant bright illumination, beyond that commonly found in natural environments, negatively affects multiple measures of immune function (Durrant *et al*., 2015; Jones *et al*., 2015). However, whether exposure to more ecologically relevant levels of ALAN has a comparable effect on cricket immune function is untested.

We investigated the effect of lifelong exposure to four different ecologically relevant levels of ALAN (0, 1, 10, or 100 lx) on lifetime adult immune function in male and female crickets. We predicted that ALAN would compromise immune function, as previously demonstrated under constant bright light (Durrant *et al*., 2015; Jones *et al*., 2015). Given recent studies highlighting that even dim ALAN (<5 lx) is capable of affecting other physiological processes including circadian rhythm (Evans *et al*., 2007; Brüning *et al*., 2015), melatonin release (Dominoni *et al*., 2013b), sleep (Aulsebrook *et al*., 2018), the timing of foraging and reproduction (Robert *et al*., 2015; Russ *et al*., 2015b), physiological condition (Raap *et al*., 2016b), survival (McLay *et al*., 2017), and immune function in vertebrates (Aubrecht *et al*., 2014), we also predict that these effects on immune function would be observed at even the lowest levels of ALAN (namely, 1 lx).

## Materials and methods

Experimental crickets were sourced from a 10th generation laboratory‐adapted population of founders captured in Victoria, Australia (37.56238 S, 145.31920 E). Stock population crickets (approximately 1000 per generation) were maintained under standard conditions, as detailed in (Durrant *et al*., 2015), and held in a climate‐controlled laboratory under a 12 h light : 12 h dark cycle.

### ALAN treatments and rearing conditions

To investigate the effect of ecologically relevant levels of ALAN on cricket immune function, we maintained experimental individuals from eggs to adults under comparable simulated daylight conditions (2600 lx; equivalent to a cloudy day) for 12 h followed by one of four ALAN treatments (0, 1, 10, or 100 lx) for a further 12 h (for further information regarding the rearing and treatment groups see, Botha *et al*., 2017; Durrant *et al*., 2018). To create the different ALAN treatments, we used retrofitted Westinghouse incubators (model number WRM4300WB‐R) set at a constant 28 °C and lit by cool‐white (6800 Kelvin during simulated day and 5900 Kelvin simulated night) LED strip lighting on the front panels (World of Thought; Melbourne, Australia). While this is a laboratory based study, the ALAN treatments were chosen to fall within the range of conditions present in and around urban areas, where 100 lx is acknowledged as an extreme but is an intensity present in a heavily urbanized city center where crickets are present (Gaston *et al*., 2013). We note that *sensu* Gaston *et al*. (2017), we use lx as our unit of light. While this measure is based on human photopic vision, and thus does not necessarily capture the relative effects of light influencing crickets *per se*, its use ensures a direct link to illuminance as commonly measured in the environment and employed in the design and mitigation of artificial lighting systems. Individual incubator effects were accounted for and mitigated by alternating the light regimen in each incubator thrice weekly, and swapping the crickets accordingly, ensuring each incubator contributed equally to each regimen, but crickets were always maintained on their designated light regimen. Crickets were also rotated within incubators so that each experimental individual experienced a range of positions in each incubator. It should also be noted that while crickets were isolated in an incubator in terms of light regimen, incubators were not sound proofed and thus no cricket was acoustically isolated from one another regardless of their ALAN treatment or incubator.

Eggs were initially maintained in family groups of comparable age (designated by the date when laid) at densities of between 3 and 25 per container (dependent on the number laid during the given 2‐d oviposition period). Within 24 h of their subsequent hatching, each first instar nymph was transferred to an individual transparent rearing container (70 × 70 × 40 mm), which contained a folded 10 × 30 mm cardboard shelter, *ad libitum* water and dried cat food (Friskies Senior; Rhodes, Australia) where they were maintained until their final juvenile moult. On the day of their final juvenile moult, each adult cricket was weighed (to the nearest mg) and transferred to a larger transparent container (150 mm length × 90 mm width × 50 mm height) with a piece of egg carton for shelter and *ad libitum* food and water. Adults were checked daily until death or until the completion of the experiment (33 ± 1 d after the final juvenile moult) at which point they were killed by freezing. To control for potential size‐related variation in immune response, following death, both femurs were removed from an individual cricket; taped to a glass slide and then digitally photographed with a Canon EOS 60D (Tokyo, Japan) mounted at 10× magnification on an Olympus SZX7 stereomicroscope (Tokyo, Japan). Mean femur length for each cricket was determined using image J (Version 1.48V, NIH; Maryland, USA).

There was significant variation in adult survival across the ALAN treatments resulting in more 100 lx juveniles surviving to the adult stage (Durrant *et al*., 2018) and, while not critical to the current experiment, to ensure families contributed equitably, we randomly selected a maximum of six individuals from each ALAN treatment and, where possible, ensured that the number of males and females per family per ALAN treatment was comparable (mean ± SE number of females per family per treatment = 2.20 ± 0.07; males = 2.55 ± 0.09).

### Haemolymph extraction for immune measures

For each individual cricket we assessed three measures of immune function at 3 ± 1, 17 ± 1, and 31 ± 1 d after the final juvenile moult, herein referred to as Week 0, Week 2, and Week 4, respectively. These three time‐points provide a baseline measure of immune function following juvenile development (Week 0) and also a longitudinal measure of adult immune function (comparisons between Week 0, Week 2, and Week 4).

To collect the haemolymph, a small puncture was made in the left side of the cricket abdomen using a 27G sterile needle (Becton Dickinson and Co.; Melbourne, VIC, Australia). The resulting wound exudes a small haemolymph bubble on the cuticle surface from which we collected a maximum of 6 μL. For haemocyte concentration, 1 μL of this haemolymph was collected using a micropipette and immediately transferred to a 0.5 mL Eppendorf tube (Sarstedt; Mawson Lakes, SA, Australia) maintained on ice, containing 20 μL of pre‐prepared anticoagulant solution (100 mmol/L NaOH, 150 mmol/L NaCl, 22 mmol/L EDTA, 45 mmol/L citric acid in distilled water). For the lytic and phenol oxidase (PO) activity assays, a further 4.5 μL of haemolymph was collected, transferred to a 0.5 mL Eppendorf tube containing 60 μL of phosphate buffer saline (PBS; 11.9 mmol/L phosphate, 137 mmol/L NaCl, 2.7 mmol/L KCl, pH 7.4), snap frozen in liquid nitrogen and then stored at −80 °C until later analysis. We note that crickets have a very efficient coagulation system, as small puncture wounds are common during fights or interactions, so crickets recovered immediately from these procedures and there were no obvious effects on survival.

### Haemocyte concentration

To determine the concentration of haemocytes, a 10 μL sample of the haemolymph‐anticoagulant solution (described above) was placed onto a Neubauer hemocytometer (Blaubrand; Wertheim, Germany) at 100× magnification on an Olympus BX50 stereomicroscope (Olympus; Tokyo, Japan). The number of haemocytes was counted and expressed as a concentration, number of cells/mL (Drayton & Jennions, 2011).

### Lytic activity

To assess lytic activity, 10 μL of the haemolymph‐PBS solution (described above) was added to a round bottom 96‐well plate (Sarstedt; Mawson Lakes, SA, Australia). Each sample was added twice, as technical duplicates. To each well, we added 80 μL of *Micrococcus lutus (lysodeikticus)* cell wall suspension (3 mg/mL PBS; Sigma‐Aldrich; North Ryde, NSW, Australia; ATCC No. 4698). Lysozymes in the haemolymph gradually degrade the bacteria in the solution causing the turbidity of the solution to decrease. Relative change in absorbance at 490 nm and 30 °C was measured over a 120‐min period in a microplate spectrometer (BioTek EL × 800 Absorbance Microplate Reader, Millenium Science; Mulgrave, Australia), with greater transparency of the solution indicating greater lytic activity. Samples were stratified across plates and each plate included negative controls of PBS only, as well as the same pooled biological quality control run on every plate. The mean intra‐ and intercoefficients of variation for all lytic assays were 13.7% and 12.3%, respectively (*n* = 19 plates).

### Phenoloxidase (PO) activity

When in circulation in the haemolymph, PO is present as an inactive precursor, pro‐phenoloxidase. Thus, to measure the total PO present, we first cleaved all pro‐phenoloxidase with the proteolytic enzyme, α‐chymotrypsin (Moreno‐Garcia *et al*., 2013). For each sample, 5 μL of the haemolymph‐PBS solution was added to a round‐bottom 96‐well plate, followed by 7 μL of 1.3 mg/mL bovine pancreas α‐chymotrypsin (Sigma‐Aldrich). After a 20‐min incubation period at room temperature we added 90 μL of L‐dihyroxyphenylalanine (L‐DOPA; Sigma‐Aldrich). The relative change in absorbance at 490 nm was measured over a 120‐min period in a microplate spectrophotometer. PO converts L‐DOPA to dopachrome causing the solution to darken. The greater change in absorbance indicated greater PO activity. As above, samples were stratified across plates and each plate included technical duplicates of each sample, negative controls of PBS only, as well as the same pooled biological control run on every plate. The mean intra‐ and intercoefficients of variation (calculated as the ratio between the standard deviation across cells within a plate [intra‐] or across plates [inter‐] to the mean of all cells or plates) for all PO assays were 6.1% and 5.7%, respectively (*n* = 19 plates).

### Statistical analyses

Statistical analyses were performed in JMP 12.1.0 (SAS Institute, NC, USA). We used generalized linear mixed models to explore the effect of ALAN on immune function throughout the adult life of the crickets. Variables were assessed for normality and haemocyte data were subsequently log_e_ transformed prior to analyses. We first assessed baseline variation across the treatment groups by differences in Week 0 measures. We then performed a second set of analyses exploring the change over time, with week as a categorical variable. ALAN treatment, sex, week, and whether they survived to the end of the experimental period (Yes or No) were included as categorical and femur length as a continuous fixed effect in all models; family ID, individual ID, and plate number were included as random effects. All biologically appropriate interactions were included. Each model was reduced using hierarchical removal of all terms with a significance of *P* > 0.1 except the main ALAN treatment. Once the minimum adequate model was obtained, excluded terms (*P* > 0.1) were reintroduced back to confirm the model fit did not improve with their inclusion. To assess correlations between the three immune measures, we used Spearman's rank correlation tests using untransformed data throughout. All tests were two‐tailed with a significance level of *P* < 0.05. Unless otherwise stated, significant interactions were assessed using *post hoc* planned contrast tests, and data presented are means ± SE. Samples sizes for all models are outlined in Table S1.

**Table 1:** Means ± standard errors (sample size) and associated generalized linear mixed outputs from models exploring the initial (week 0 after final moult) effect of lifelong ALAN exposure (0, 1, 10, and 100 lx) Ln (haemocyte concentration in cells/mL × 10^6^) (A, D), change in lytic activity (B, E) and change in phenoloxidase (PO) activity (C, F) for females and males respectively. Family and assay plate number were included as random terms in all models. Significant P‐values highlighted in bold

Model parameters	0 lx	1 lx	10 lx	100 lx	Statistic	*P* value
Female						
(A) Ln (haemocyte concentration)	2.89 ± 0.14 (22)	2.77 ± 0.13 (27)	2.78 ± 0.14 (26)	2.60 ± 0.12 (26)	*F*_3,77.03_ = 1.59	0.20
(B) Lytic activity (Δ absorbance)	0.27 ± 0.03 (17)	0.37 ± 0.04 (23)	0.28 ± 0.03 (21)	0.31 ± 0.02 (24)	*F*_3,78.05_ = 1.66	0.18
(C) PO activity (Δ absorbance)	0.75 ± 0.10 (18)	0.55 ± 0.06 (23)	0.44 ± 0.05 (22)	0.56 ± 0.08 (25)	*F*_3,81.56_ = 2.15	0.10
Male						
(D) Ln (haemocyte concentration)	2.69 ± 0.13 (27)	2.58 ± 0.09 (30)	2.69 ± 0.12 (27)	2.61 ± 0.10 (35)	*F*_3,95.1_ = 0.25	0.86
(E) Lytic activity (Δ absorbance)	0.24 ± 0.03 (21)	0.26 ± 0.02 (27)	0.31 ± 0.03 (27)	0.30 ± 0.02 (32)	*F*_3,95_ = 1.80	0.16
(F) PO activity (Δ absorbance)	0.24 ± 0.03 (21)	0.28 ± 0.03 (27)	0.30 ± 0.03 (28)	0.30 ± 0.03 (32)	*F*_3,89.62_ = 0.94	0.42

## Results

### Baseline Week 0 measures

Baseline measures at Week 0 were comparable for all treatments and both sexes (Table 1A–F; *P* > 0.10 for all analyses). There was significant variation across families for most immune measures (percentage of family level variation for female haemocytes counts = 18.8%, lytic activity = 5.3%, PO activity = 0.0%; and for male haemocytes = 9.4%, lytic activity = 14.4%, PO activity = 10.3%).

### Longitudinal variation in immune function (Week 0 to Weeks 2 and 4)

#### Haemocyte concentration

**Figure 1:**
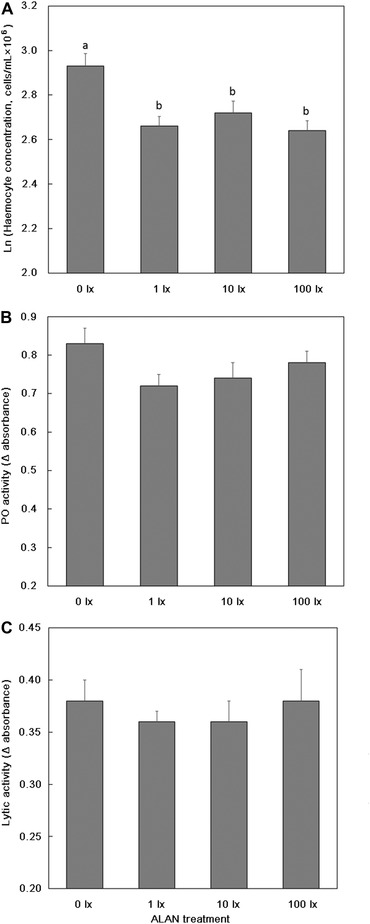
Variation across ALAN treatments in (A) heamocyte concentration, (B) lytic activity, and (C) phenoloxidase (PO) activity for adult crickets reared from egg through to adults under night‐time light leavels (ALAN treatment) of 0, 1, 10, and 100 lx. Data presented are means ± SE; different letters, where present, denote significant (*P*< 0.05) differences between ALAN treatments.

**Figure 2:**
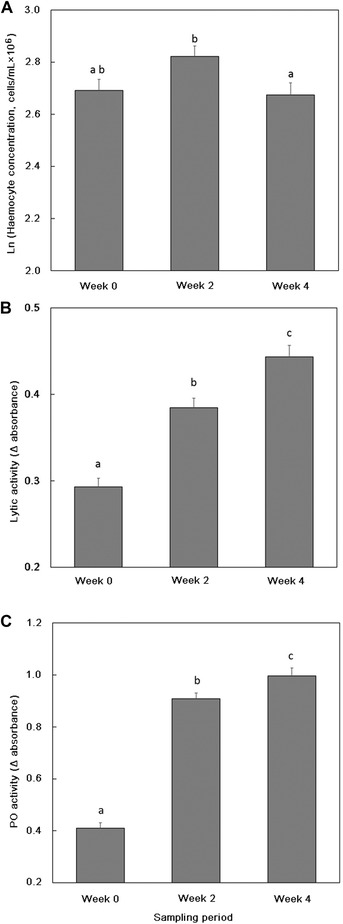
Variation in (A) haemocyte concentration (B) lytic activity, and (C) phenoloxidase (PO) activity for adult cricket at Week 0, Week 2, and Week 4 means ± SE; different letters, where present, denote significant (*P*< 0.05) differences between treatments.

Haemocyte concentrations (cells/mL × 10^6^) varied with ALAN treatment (*P* = 0.0003; Table 2A; Fig. 1A), sampling week (*P* = 0.04; Table 2A; Fig. 2A) and were greater for females (mean ± SE = 2.83 ± 0.04) compared to males (mean ± SE = 2.65 ± 0.03; *P* = 0.0002; Table 2A). *Post hoc* analyses revealed crickets in the 1, 10, and 100 lx ALAN treatments had lower haemocyte concentrations compared to 0 lx crickets (Table 2A) and, on average, haemocyte concentrations increased marginally between the Week 0 and Week 2 samples and then declined at the Week 4 sample (Fig. 1A). There was also significant variation between families (percentage variation explained by the random family term = 9.1%, *P* = 0.05; Table 2A).

**Table 2:** Mixed models exploring the effect of lifelong ALAN treatment (0, 1, 10, and 100 lx) over time (Weeks 0, 2, and 4 after final moult) for (A) haemocyte concentration (cells/mL × 10^6^), (B) lytic activity (Δ absorbance), and (C) phenoloxidase (PO) activity (Δ absorbance). Family, individual ID, and assay plate number were included as random terms in all models. Significant P‐values highlighted in bold

Model parameters	Statistic	*P* value
(A) Haemocyte concentration (cells/mL × 10^6^) (Ln transformed)		
ALAN treatment	*F*_3,205.3_ = 6.48	**0.0003**
Week	*F*_2,358.5_ = 3.32	**0.04**
Sex (F > M)	*F*_1,205.2_ = 14.40	**0.0002**
Week × sex	*F*_2,392.4_ = 2.640	0.07
Family (variation = 9.1%)		**0.05**
Individual (variation = 4.5%)		0.31
Assay plate number (variation = 1.6%)		0.32
(B) Lytic activity (Δ absorbance)		
ALAN treatment	*F*_3,201.3_ = 1.86	0.14
Week	*F*_2,342.5_ = 51.70	**<0.0001**
Sex	*F*_1,200.6_ = 0.56	0.56
ALAN treatment × Week	*F*_6,351.9_ = 2.90	**0.008**
Week × Sex	*F*_2,357.3_ = 7.04	**0.001**
Femur length (mm)	*F*_1,210.2_ = 3.45	0.06
Family (variation = 12.1%)		0.06
Individual (variation = 24.1%)		**<0.0001**
Assay plate number (variation = 2.1%)		0.19
(C) PO activity (Δ absorbance)		
ALAN treatment	*F*_3,217.5_ = 0.38	0.76
Week	*F*_2,398.1_ = 204.20	**< 0.0001**
Sex (F > M)	*F*_1,219_ = 83.10	**<0.0001**
Survived (Y, N)	*F*_1,307.9_ = 15.70	**<0.0001**
Family (variation = 0.4%)		0.36
Individual (variation = 10.7%)		**0.03**
Assay plate number (variation = 5.0%)		0.07

#### Lytic activity

**Figure 3:**
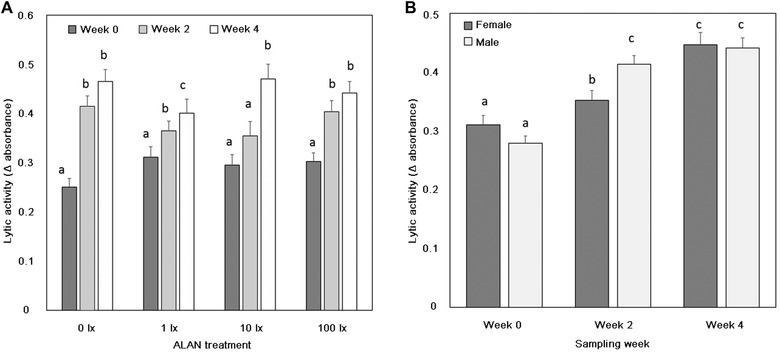
Variation in lytic activity between sampling week and (A) ALAN treatment and (B) sex. Data presented are means ± SE different letters, where present, denote significant (*P* < 0.05) differences between weeks for Fig. 3A and males and females for Fig. 3B.

Patterns of lytic activity were more complex. There was no main effect of ALAN treatment (*P* = 0.14; Table 2B, Fig. 1B) or Sex (*P* = 0.56) on lytic activity. However, lytic activity increased across the three sampling periods (*P* < 0.0001; Fig. 2B). This latter relationship was driven by significant interactions between ALAN treatment and Week (*P* = 0.008; Fig. 3B) and also between Sex and Week (*P* = 0.001; Fig. 3B). *Post hoc* analyses revealed that lytic activity typically increased over the three sampling periods and was thus higher at the Week 4 compared to the Week 0 sampling period for all ALAN treatments, but there was variation with respect to the amount of increase at the Week 2 sampling period (Fig. 3A). Similarly, lytic activity was comparable for males and females at Week 0 and Week 4, but males had higher lytic activity than females in Week 2 (Fig. 3B). There was considerable individual variation in lytic activity (percentage variation explained by the random term individual = 24.1%, *P* < 0.0001).

#### Phenoloxidase (PO) activity

PO activity was comparable across the ALAN treatments (*P* = 0.76; Table 2C, Fig. 1C) but it increased significantly across the three sampling periods (*P* < 0.0001; Fig. 2C). It was significantly greater for females (mean ± SE = 0.90 ± 0.02) compared to males (mean ± SE = 0.65 ± 0.02; *P* < 0.0001) and was higher in individuals that survived to the end of the experiment (mean ± PO for individuals that died = 0.56 ± 0.04 (*n* = 48); survived = 0.80 ± 0.02 (*n* = 183); *P* < 0.0001). There was considerable individual variation in PO activity (percentage variation explained by the random term individual = 10.7%, *P* = 0.03).

#### Correlations between immune parameters

Haemocyte concentration was positively correlated to lytic activity for males and females across all weeks (all *P* < 0.05) but was unrelated to PO activity (All *P* > 0.1, Table 3). In contrast, lytic activity was correlated with PO activity for Weeks 0 and 4 but unrelated at Week 2.

**Table 3:** Spearman's rank correlations (*r_s_*) between haemocyte concentrations, lytic activity, and phenoloxidase (PO) activity for females and males across the three sampling periods (Week 0, Week 2, and Week 4)

	Week 0		Week 2		Week 4	
	Lytic activity	PO activity	Lytic activity	PO activity	Lytic activity	PO activity
Female						
Haemocyte	**0.24** *	0.16	**0.32** **	0.13	**0.44** ***	0.13
Lytic activity	–	**0.21** *	–	0.05	–	**0.29** *
Male						
Haemocyte	**0.38** ***	0.16	**0.36** **	0.01	**0.35** **	0.03
Lytic activity	–	**0.35** **	–	0.07	–	**0.36** **

Significant *r_s_* values shown in bold; ^*^ *P* < 0.05, ^**^ *P* < 0.01, and ^***^ *P* < 0.0001.

## Discussion

Our experiment yielded several key findings related to the impact of dim light at night on immune function. First baseline levels of adult immune function were comparable regardless of sex or juvenile rearing treatment. Second, following a wounding challenge (in the Week 0 sampling period), adults reared under the presence of artificial light at night were less able to upregulate their haemocytes and thus had a potentially reduced core cellular defense pathway compared with crickets reared under 0 lx at night. We found a correlation between haemocyte count and lytic activity but neither lytic activity nor PO activity varied in response to the presence of ALAN, although they did increase across the three sampling periods. Finally, there was little concordance across the immune parameters measured with respect to their lifetime response to the presence of ALAN, however our data are in alignment with previous data in *T. commodus* using significantly higher night‐time lx levels (Durrant *et al*., 2015; Jones *et al*., 2015). Critically, our data suggest that the intensity of nighttime lighting required to generate shifts in immune parameters (and particularly haemocyte concentrations) in the field cricket are several orders of magnitude lower than previously reported.

These data concur with a growing number of studies demonstrating that even low levels of ALAN can generate shifts in life history traits of many species, including immune parameters (Bruning *et al*., 2011; Dominoni *et al*., 2013c; Dominoni *et al*., 2014; van Geffen *et al*., 2014; Brüning *et al*., 2015; Dominoni, 2015; van Geffen *et al*., 2015a; van Geffen *et al*., 2015b; McLay *et al*., 2017; van Langevelde *et al*., 2017; Durrant *et al*., 2018). Our longitudinal approach offers some additional insights into immune responses in *T. commodus*. First, the fact that all initial responses were comparable regardless of immune assay, ALAN treatment or sex suggests either that ALAN has little impact on juvenile immune function (potentially because conditions were otherwise so benign) or that strong selection on the juvenile phase has resulted in only the highest quality individuals surviving through to adults. The increased upregulation of haemocytes observed for 0 lx crickets suggests a strong capacity to respond to future challenges which is lacking when crickets are exposed to even relatively low levels of ALAN. While few studies have explored the effects of low levels of ALAN on immune function in invertebrates, similar effects on analogous cellular components of the immune system in vertebrates are reported (Bedrosian *et al*., 2011b; Aubrecht *et al*., 2014; Ikeno *et al*., 2014).

The effects of ALAN on lytic and PO activity were less pronounced than that of haemocytes. Our data suggest that ALAN may have some effect on the degree of lytic response, as indicated by the differences across the treatments with respect to how rapidly they changed at the Week 2 sample, but these were somewhat more complex than those identified for haemocyte concentration and were highly variable between individuals. Nonetheless, assuming differences in haemocyte concentrations and potentially lytic activity translate into a reduction in immune function, this could then result in significant reductions in the overall fitness of individuals living in urban environments (Ryder & Siva‐Jothy, 2001; Adamo, 2004a, b; Schmid‐Hempel, 2005).

Given the above, it may seem surprising that haemocyte concentration and lytic activity were not correlated with adult survival probability in this experiment, as seen in Table 3. However, the reason may be a product of the experimental design. The relatively benign laboratory conditions, *ad libitum* food and water and an absence of any disease or parasite risk, means the greatest immunological threat to the survival of these crickets was the repeated wounding events for the extraction of haemolymph. The component of a cricket immune system that is primarily responsible for responding to such an event is phenoloxidase (Kanost & Gorman, 2008), and indeed, a particularly large upregulation of PO activity was determined at Week 2, following the initial wounding event. Furthermore, individuals who survived until the end of the experiment had significantly higher PO activity than those that did not survive but this was unrelated to the presence of ALAN. Future studies could try to elucidate some of the potential fitness consequences of ALAN by either imposing additional stressors in a laboratory setting or conducting experiments in a semi‐natural scenario, where there is lower food availability, increased risk of parasitic load and thus, a greater variety of immune challenges. For nocturnal species, such as *T. commodus*, the consequences of a reduced immune capacity are likely to be far greater under natural conditions and will most likely impact their overall survival and reproductive success (Rolff & Siva‐Jothy, 2003).

Our data were unable to elucidate the underlying physiological mechanism driving the observed variation in immune function. While wounding alone imposes a significant immune challenge promoting upregulation of the immune system we are unable to exclude age‐related changes as a possible interacting factor (Adamo *et al*., 2001; Park *et al*., 2011; Pinera *et al*., 2013). Experimental manipulation of the timing of initial wounding would further our understanding in this context. Similarly, the ALAN related changes in haemocyte concentrations are likely to be linked to the light sensitive melatonin pathway. The indolamine melatonin is a key regulator of circadian rhythm and is a powerful antioxidant found in both vertebrates and invertebrates (Poeggeler, 1993; Vivien‐Roels & Pevet, 1993; Tan *et al*., 2010). Exposure to light at night suppresses melatonin production in vertebrates (Dominoni *et al*., 2013b; Brüning *et al*., 2015; Robert *et al*., 2015) and invertebrates including *Teleogryllus commodus*. Several experiments, including our own in *T. commodus*, report a link between melatonin and the cellular immune response (Demas & Nelson, 1998; Drazen & Nelson, 2001; Raghavendra *et al*., 2001; Jones *et al*., 2015). Moreover, we have previously demonstrated that dietary supplementation with melatonin can mitigate the negative effects on immune function of exposure to even extremely bright light (Jones *et al*., 2015). Further work is required to assess whether a comparable mechanism operates at lower intensities of ALAN as is observed for other species (Dominoni *et al*., 2013b; Brüning *et al*., 2015; Robert *et al*., 2015) but, given its ubiquity across the animal kingdom and its largely comparable modes of action, it seems highly likely this that it is acting as a key mechanistic link in the observed effects of ALAN on immune function.

It is likely that further insights could be gained from a multigenerational study that could also assess the transgenerational effects of ALAN. The large amount of family and individual variation demonstrated in this experiment, particularly for lytic and PO activity, suggest that there is likely a genetic component to an individual's response to ALAN. This variability may also explain the lack of differences between ALAN treatments. To date no study has specifically explored heritable variation in species resilience in relation to the presence of ALAN but there is mounting evidence suggesting variation in the genetic structure of urban v rural populations (Evans, 2010; Hopkins *et al*., 2018), which makes this highly likely.

Our study demonstrates that low levels of ALAN have a negative effect on haemocyte concentration. Furthermore, correlations identified between aspects of the immune system hint toward a causal mechanistic link between ALAN and reductions in biological fitness, with a substantial degree of familial variation potentially being a factor to mask a strong correlation. Perhaps more critically, the effects of lifelong exposure to ALAN of 1 lx are comparable to those of 100 lx. Hence the effects of ALAN could be large and widespread, particularly in more malign conditions such as those of the natural environment. Understanding the physiological impacts of ALAN, as well as why and how they are occurring and to what extent they will impact individual fitness, is crucial to inform the management of this growing environmental concern.

## Supporting information


**Table S1**. Sample sizes used for statistical models.
