# Supplementary material for: Dim artificial light at night reduces the cellular immune response of the black field cricket, Teleogryllus commodus
Source: Insect Sci. 2019 Mar 7;27(3):571–82. doi: 10.1111/1744-7917.12665 (PMC7277038; doi:10.1111/1744-7917.12665)
Supplement: Supplementary file 1 — Table S1. Sample sizes used for statistical models. [file INS-27-571-s001.docx]

**Supplementary Table 1.** Sample sizes used for statistical models.

| **Sex** | **Light treatment** | **Week** | **Total Crickets** | **Survived** | **Haemocyte Assay** | **Lysozyme Assay** | **PO Assay** |
| --- | --- | --- | --- | --- | --- | --- | --- |
| Female | 0 lx | 0 | 22 | 20 | 22 | 17 | 18 |
|  |  | 2 |  |  | 22 | 21 | 22 |
|  |  | 4 |  |  | 19 | 19 | 19 |
|  | 1 lx | 0 | 27 | 17 | 27 | 23 | 23 |
|  |  | 2 |  |  | 22 | 24 | 24 |
|  |  | 4 |  |  | 18 | 16 | 16 |
|  | 10 lx | 0 | 28 | 22 | 26 | 21 | 22 |
|  |  | 2 |  |  | 24 | 24 | 25 |
|  |  | 4 |  |  | 19 | 17 | 17 |
|  | 100 lx | 0 | 29 | 23 | 26 | 24 | 25 |
|  |  | 2 |  |  | 26 | 26 | 27 |
|  |  | 4 |  |  | 23 | 23 | 23 |
| Male | 0 lx | 0 | 28 | 24 | 27 | 21 | 21 |
|  |  | 2 |  |  | 24 | 22 | 23 |
|  |  | 4 |  |  | 22 | 21 | 21 |
|  | 1 lx | 0 | 31 | 24 | 30 | 27 | 27 |
|  |  | 2 |  |  | 27 | 26 | 26 |
|  |  | 4 |  |  | 24 | 25 | 24 |
|  | 10 lx | 0 | 30 | 22 | 27 | 27 | 28 |
|  |  | 2 |  |  | 27 | 25 | 25 |
|  |  | 4 |  |  | 21 | 19 | 20 |
|  | 100 lx | 0 | 36 | 31 | 35 | 32 | 32 |
|  |  | 2 |  |  | 35 | 32 | 33 |
|  |  | 4 |  |  | 33 | 32 | 32 |
